# Efficient Polarization Beam Splitter Based on All-Dielectric Metasurface in Visible Region

**DOI:** 10.1186/s11671-019-2867-4

**Published:** 2019-01-25

**Authors:** Jing Li, Chang Liu, Tiesheng Wu, Yumin Liu, Yu Wang, Zhongyuan Yu, Han Ye, Li Yu

**Affiliations:** 1 State Key Laboratory of Information Photonics and Optical Communications, Beijing University of Posts and Telecommunications, Beijing, 100876 China; 2 School of Science, Beijing University of Posts and Telecommunications, Beijing, 100876 China; 3 College of Information and Communication Engineering, Guilin University of Electronic Technology, Guilin, 541004 China

**Keywords:** Phase shift, Metasurface, Polarization beam splitters, Refraction, Visible region

## Abstract

In this paper, we present an all-dielectric gradient metasurface, composed of periodic arrangement of differently sized cross-shaped silicon nanoblocks resting on the fused silica substrate, to realize the function of polarization split in visible region. The cross-shaped silicon block arrays can induce two opposite transmission phase gradients along the *x*-direction for the linear *x*-polarization and *y*-polarization. By properly designing, the metasurface can separate the linearly polarized light into *x*- and *y*-polarized ones, which propagate at the same angle along the left and right sides of the normal incidence in the *x*-*z* plane. Particularly, when a beam with the polarization angle of 45.0° is incident on the proposed device, the *x*- and *y*-polarized transmitted ones possess nearly equal intensity within the wavelength range from 579 to 584 nm. We expect the proposed polarization beam splitter can play an important role for future free-space optical devices.

## Introduction

In recent years, metasurfaces, two-dimensional subwavelength structures composed of nanoantennas in an array configuration, have obtained enormous attentions. Metasurface can manipulate the incident light on a subwavelength scale because its ultrathin structured thickness introduces abrupt changes of the incident beam parameters. For example, the phase [1–5], amplitude [6–9], and polarization [10–13] of the incident beams can be manipulated by adjusting the shape, size, and orientation of the subwavelength nanoantennas. In comparison with the conventional bulky materials, the metasufrace devices are easier to be fabricated and their ultrathin thickness in the optical path can greatly suppress transmission losses. Based on the above exciting advantages, metasurfaces have been used in many applications, such as polarization converter [11–13], full-color printing [14], holography [15], flat lenses [16], optical vortex generation [4, 17], and spectrum splitting [18–21].

Metallic nanostructures were utilized to constitute metasurfaces with beam deflection originally [1, 22, 23]. The required 2π phase coverage can generally be achieved based on two methods. The one is generating two independent resonances, each of which introduces a phase shift of π. The other is to spatially rotate the polarization-dependent subwavelength resonators from 0° to 180°. However, the absorption losses of metallic metasurfaces limit the efficiency in transmission mode. All-dielectric metasurfaces have recently been proposed to substitute the metallic ones due to their low absorption losses [24–28]. To date, three different approaches have been demonstrated to realize the 2π phase shift in the all-dielectric metasurfaces, geometric phase [27], Mie resonance [2, 4, 7], and Fabry–Pérot resonance [3, 28]. The first method is similar to the above second way of metallic metasurface; it works for circularly polarized light. The second mechanism covers the full 2π phase range based on spectrally overlapping magnetic and electric resonances; the metasurface designed based on this way is also known as Huygens metasurface. The third method, just as the one utilized in this paper, uses high aspect ratio nanoantennas to obtain the desired phase control. The antennas can be considered as truncated waveguides in this case, and transmission phase is manipulated by the effective refractive index of the fundamental mode in differently sized dielectric antennas. Silicon is generally applied in all-dielectric metasurface devices [2–4] for its high refractive index, low loss, and mature process manufacturing. As for some other low refractive index materials, such as silica (SiO_2_), silicon nitride (Si_3_N_4_), and titanium dioxide (TiO_2_), their losses may be ignored, but the higher aspect ratios make the fabrication very challenging.

Polarization beam splitter, a device which can separate an optical beam into two orthogonally polarized components propagating along different paths, is an important component in optical systems. Polarization beam splitters reported in the literatures are designed primarily based on the following structures, including subwavelength structures [29–31], hybrid plasmonic couplers [32], gratings [33], multimode interference (MMI) structures [34], and asymmetrical directional couplers [35, 36]. Farahani and Mosallaei [29] proposed an infrared reflectarray metasurface to reradiate incoming light into two orthogonally polarized reflective beams. Guo et al. [30] designed a polarization splitter based on silicon metasurfaces at the specific wavelength of 1500 nm. In this work, we propose a simple and large-angle deflected polarization beam splitter based on dielectric metasurface, which is constructed by different cross-shaped silicon resonator arrays atop of the silica substrate. When *x*- or *y*-polarized light is normally incident, the polarization direction of the transmitted light is the same as that of the incident light. At a wavelength of 583 nm, the deflected angle is 46.78° and the deflection efficiency is 63.7% under *x*-polarized incidence, while the deflection efficiency is 66.4% and the deflected angle is − 46.78° for *y*-polarized one. Furthermore, the proposed device is capable of separating the linearly polarized light into *x*- and *y*-polarized ones. Especially, when the polarization of the incident light is at an angle of 45° to the *x*-axis, two orthogonally polarized transmitted beams possess approximately equal intensities within the wavelength region from 579 to 584 nm.

## Methods

**Fig. 1:**
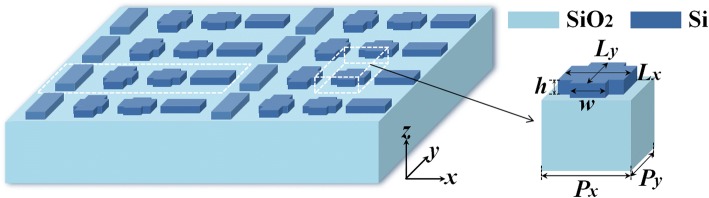
Schematic configuration of the proposed cross-shaped metasurface acting as a polarization beam splitter

Figure 1 schematically depicts the configuration of the proposed polarization beam splitter device, which is designed based on an all-dielectric metasurface. The metasurface is composed of an array of cross-shaped silicon blocks placed on the silica substrate. The optical constants of silicon are taken from Ref [37], and the refractive index of silica is 1.45. The silicon block height *h* is set as 260 nm; the period of the unit cell along the *x*- and *y*-directions are optimized to be *Px* = 200 nm and *Py* = 200 nm. The numerical simulation is performed by three-dimensional finite-difference time-domain (FDTD) models, in which periodic boundary conditions are applied in the both *x*- and *y*-directions and perfectly matched layers are used along the *z*-direction. The plane wave is normally incident from the underneath of the substrate. The cross-shaped silicon nanoblocks array can be viewed as composed of two perpendicular silicon block arrays. One array is that the lengths *w* of the antennas along the *x*-axis remain constant while the lengths *Ly* along the *y*-axis change to induce the phase gradient under *y*-polarized incidence. On the contrary, another one introduces the phase gradient for *x*-polarized illumination by varying the lengths *Lx* of the antennas along the *x*-direction and maintaining the lengths *w* along the *y*-axis constant.

**Fig. 2:**
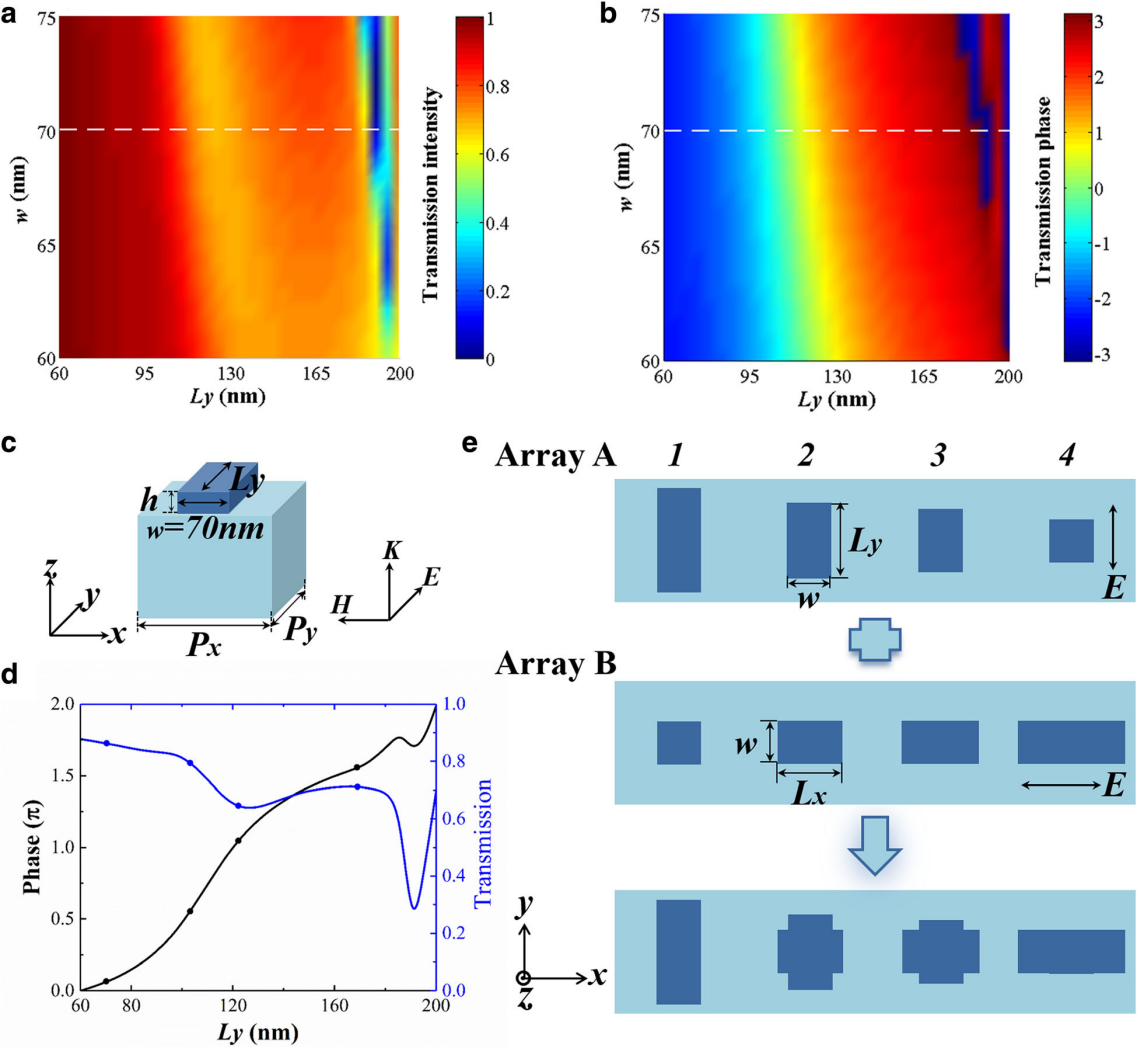
Design of the metasurface. **a** Transmission and **b** phase response as a function of width *w* and length *Ly* at a wavelength of 583 nm*.* **c** One unit of metasurface for *y*-polarized incidence. **d** Transmission and phase response of the periodic nanoblocks with widths of 70 nm as a function of the length *Ly*. **e** The design procedure of the proposed polarization beam splitter metasurface (vertical view). Here, we sort the units from left to right as unit1, unit 2, unit 3, and unit 4

Firstly, we design the phase gradient array under *y*-polarized incidence. As depicted in Fig. 2a and b, we calculate the transmission and phase response of the periodic silicon blocks by changing the width *w* from 60 to 75 nm and the length *Ly* from 60 to 200 nm at the wavelength of 583 nm. A complete 2π phase coverage cannot be obtained when the width *w* is less than 61.5 nm, but the transmission intensity decreases as the width *w* increases. Considering the manufacturing of the process, meanwhile, the width *w* of the elementary unit is fixed as 70 nm, and the length *Ly* is varied to provide the full 2π transmission phase control as depicted in Fig. 2c. The transmission and phase response as a function of the length *Ly* at the wavelength 583 nm are depicted in Fig. 2d. For large splitting angle, four different units are selected to span the 0 to 2π phase range, the lengths *Ly* of four elements are *Ly*_1_ = 169 nm, *Ly*_2_ = 122 nm, *Ly*_3_ = 103 nm, and *Ly*_4_ = 70 nm, respectively. According to the generalized Snell’s law, the angle of anomalous refraction *θ*_t_ can be obtained by the formula,

1 $$ {n}_{\mathrm{t}}\sin {\theta}_{\mathrm{t}}-{n}_{\mathrm{i}}\sin {\theta}_{\mathrm{i}}=\frac{\lambda_0}{2\pi}\frac{d\Phi}{dx} $$

where *n*_t_ and *n*_i_ are the refractive index of the transmitted and incident medium, respectively, *θ*_i_ is the angle of incidence, *λ*_0_ is the incident wavelength in vacuum, *dx* and *dϕ* are the distance and phase difference between neighboring units along the *x*-direction. In our case, the value of *dϕ* is − π/2 for *y*-polarized incidence, which is achieved by gradually decreasing the lengths *Ly* of the nanoblocks along the *x*-positive direction, as array A depicted in Fig. 2e. In order to realize the function of polarization split, the phase difference *dϕ* is set to π/2 under *x*-polarized incidence. Here, the lengths *Lx* of four units along the *x*-positive direction are 70 nm, 103 nm, 122 nm, and 169 nm, respectively, while the widths *w* keep the same value 70 nm, as array B shown in Fig. 2e. Finally, the above two arrays are combined into one cross-shaped array to form polarization beam splitting metasurface, and array A and B exhibit the phase gradients for *y*- and *x*-polarized incident light, respectively.

## Results and Discussions

**Fig. 3:**
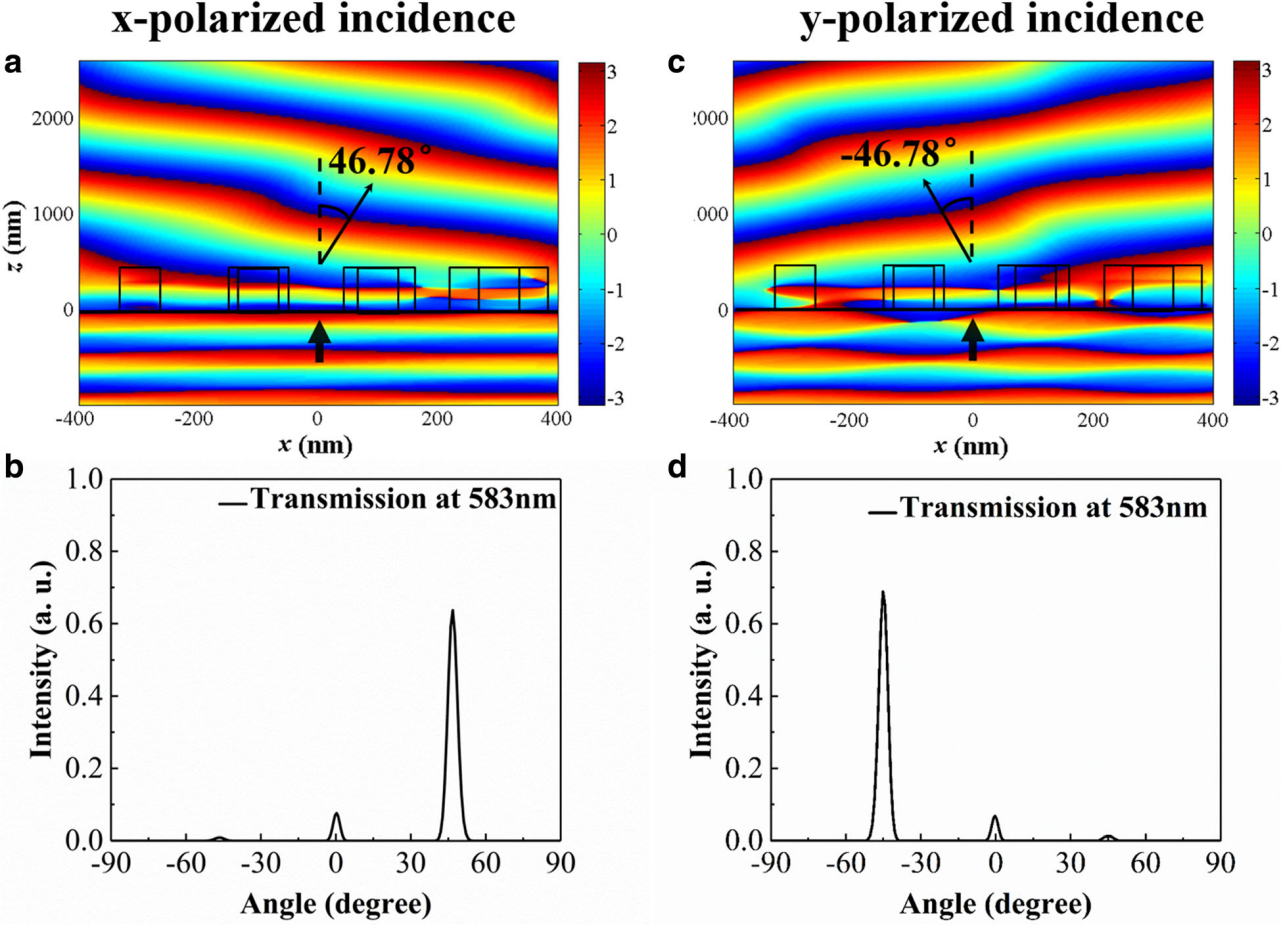
The electric field distributions near the metasurface in the *x-z* plane under **a** *x*-polarized and **c** *y*-polarized incidence. Normalized far-field intensity distributions for **b** *x*-polarized and **d** *y*-polarized normally incident light. The operating wavelength is 583 nm, and the transmitted angle is defined as positive (negative) value in the right (left) side of the normal

The optical performance of the cross-shaped metasurface acting as polarization beam splitter is simulated by three-dimensional FDTD method. In our case, the value of *dx* is 200 nm, *dϕ* is π/2, −π/2 for *x*- and *y*-polarized incidence respectively. According to the Eq. (1), the anomalous transmitted beam is deflected at an angle of 46.78° under *x*-polarized normal incidence at a wavelength of 583 nm. The transmitted electric field distribution under *x*-polarized illumination in the *x-z* plane is depicted in Fig. 3a. The observed diffraction angle 46.78° from the wavefront profile is consistent with the theoretical result. The simulated result in Fig. 3b shows that the normalized intensity in the far-field under *x*-polarized incidence. The total transmission efficiency is 69.7%, and the deflection efficiency is 63.7%, which is mainly caused by interface reflectivity (12.5%), the absorption of silicon (17.8%), and other diffraction orders (6%). Here, the deflection efficiency is defined as intensity of the deflected beam in the desired diffraction order (+ 1, − 1 order for *x*- and *y*-polarized incidence) normalized to total incident intensity. When the linear *y*-polarized light is normally incident, the electric field and normalized far-field intensity distributions at the wavelength of 583 nm are given in Fig. 3c and d, respectively. The deflected angle is − 46.78° and the corresponding deflection efficiency is 66.4%, while the total transmission efficiency is 75.2%. The reflection may be mainly caused by the high refractive index of silicon and backward scattering from edge, and the intrinsic loss of silicon in visible region leads to the high absorption. If the absorption losses are not considered in our case, the total transmission efficiencies can achieve about 90% for the above two incidences, which are comparable to the values in Ref [30]. The deflected angle is dependent on many parameters according to the Eq. (1), so it can be manipulated to satisfy our needs by adjusting the parameters, such as the period along the phase gradient direction, the operating wavelength, and another ones.

**Fig. 4:**
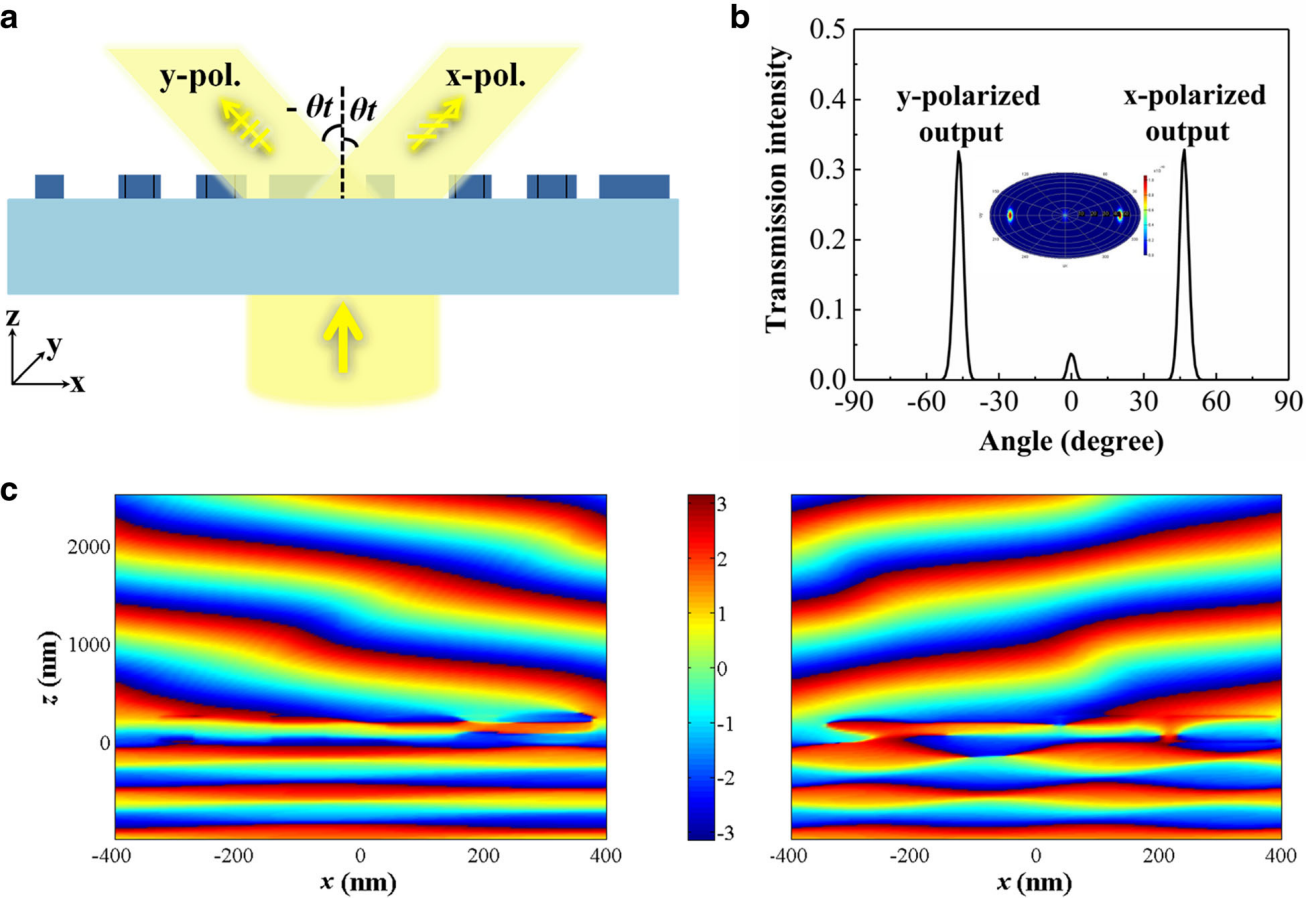
**a** Working mechanism of the proposed polarization beam splitter device (front view). **b** Normalized far-field intensity. **c** The extracted transmitted *x*-polarized (left) and *y*-polarized (right) electric field distributions of the designed metasurface under the normal incidence of 45° polarized light at the wavelength of 583 nm

A linearly polarized plane wave (*E*) can always be decomposed into two orthogonal components (*Ex* and *Ey*), which simultaneously excite two independent resonance fields in *x*- and *y*-directions. Therefore, when a linearly polarized plane wave is normally incident on the metasurface, it can be resolved into *x-* and *y*-polarized ones, which can induce opposite phase gradients along the *x*-direction. Figure 4a depicts that the working mechanism diagram of the proposed polarization beam splitter, the incident beam will be divided into *x*- and *y*-polarized ones, the corresponding deflected angles are *θ*_*t*_ and − *θ*_*t*_, which are determined by operating wavelength. The intensities of two transmitted signals are determined by the polarized angle of the incident light. When the polarization of the incident light is at an angle of 45° to the *x*-axis, the *x*- and *y*-polarized transmitted electric field distributions extracted from the total transmitted field as depicted in Fig. 4c, which also confirms the polarization splitting function of this proposed device. The normalized far-field intensity distribution for operating wavelength 583 nm is depicted in Fig. 4b; the intensity of two output beams is the same value 0.336. The total transmission intensity *I*_out_ is 0.726, so efficiencies of the total output light deflected into the + 1 diffraction order (*x*-polarization) and − 1 order (*y*-polarization) are both 46.3%. Here, the intensity of the 0 diffraction order accounts for 7.4% of the total transmission, which can be suppressed by further optimizing the geometric parameters or shapes. Furthermore, *x*- and *y*-polarized transmitted light beams possess nearly equal intensities (∣*I*_*x* − *pol*._ − *I*_*y* − *pol*._ ∣ /*I*_*x* − *pol*._ < 2%) when the polarization angle is 45° within wavelength range from 579 to 584 nm. Corresponding deflected angles and transmission intensities at different wavelengths are given in Table 1.

**Table 1:** Optical parameters at different wavelengths

Wavelength (nm)	579	580	581	582	583	584
*θ*_*t*_	46.37°	46.47°	46.57°	46.68°	46.78°	46.89°
*I*_x-pol._	0.316	0.323	0.327	0.332	0.336	0.339
*I*_y-pol._	0.313	0.320	0.327	0.332	0.336	0.339
*I*_out_	0.671	0.689	0.702	0.716	0.726	0.734

**Fig. 5:**
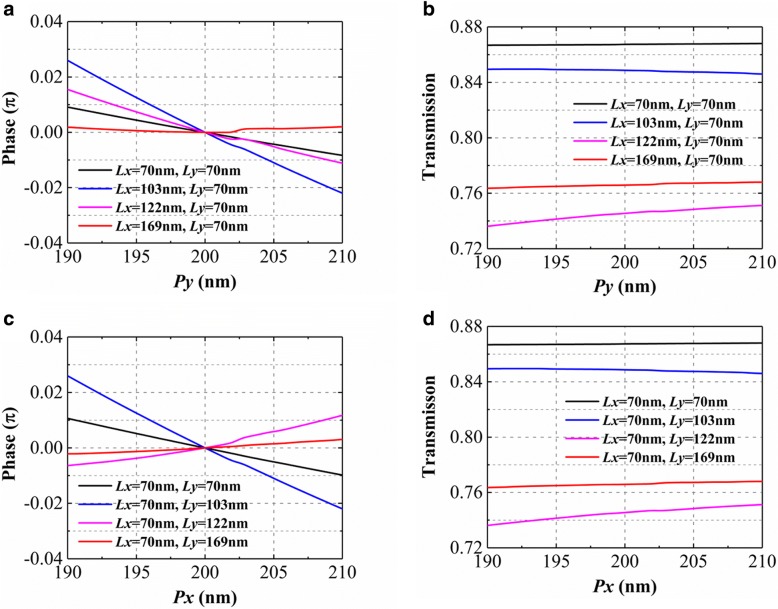
The phase response and transmission as the functions of the period in *y*(*x*)-direction when the *x*(*y*)-polarized light is incident on the uniform metasurfaces constructed by the units 1, 2, 3, and 4 of array B(A), respectively. **a** phase response and **b** transmission as the functions of *Py*. **c** phase response and **d** transmission as the functions of *Px*

In the above design process, we ideally assume that the phase and transmission response at *x*(*y*)*-*polarized incidence are not affected by the period in *y*(*x*)-direction. To prove it, we analyze the influence of the period in the *y*(*x*)-direction on the phase and transmission when the *x*(*y*)-polarized light is incident on the uniform metasurfaces constructed by the units 1, 2, 3, and 4 in array B(A), respectively. Figure 5 a and b depict that when the period *Py* in the *y*-direction varies from 190 to 210 nm, the phase changes of four types of metasurfaces are always less than 0.05π and the transmissions have almost no changes under *x*-polarized incidence. The same phenomenon occurs when the period *Px* in the *x*-direction varies from 190 to 210 nm under *y*-polarized incidence as shown in Fig. 5c and d. We think that the phase response and transmission under *x*(*y*)*-*polarized incidence are almost independent of the period in *y*(*x*)-direction in this case. Therefore, our design process is perspicuous and the method is obviously simple. In Ref [30], in order to introduce two opposite transmission phase gradients for the linearly *x*-polarization and *y*-polarization along the *x*-direction, geometric parameters of unit, width, and length are simultaneously selected by calculating phase response changing with the two parameters under the *x* and *y* linearly polarized incidence. There are no definite rules for the selection of the width and length of the units.

## Conclusions

In summary, we design a polarization beam splitter based on the all-dielectric metasurface in visible region. The metasurface is composed of cross-shaped silicon nanoblock arrays placed on top of silica dielectric substrate. When the incident light is polarized at the angle 45° relative to *x*-direction, identical intensities of the *x*- and *y*-polarized output signals are 0.336 at the operating wavelength 583 nm, which accounts for 46.3% of the total transmission intensity. Moreover, the proposed device exhibits equal-power polarization beam splitting performance for 45° polarized incidence within the wavelength region from 579 to 584 nm. We expect the polarization beam splitter can be further applied in the future all-optical integrated devices.

## Abbreviations

*dx*: Distance between neighboring units along the *x*-direction
*dϕ*: Phase difference between neighboring units along the *x*-direction
FDTD: Finite-difference time-domain
*I*_out_: Total transmission intensity
*I*_x-pol *.*_: Intensity of *x*-polarized transmitted beam
*I*_y-pol._: Intensity of *y*-polarized transmitted beam
MMI: Multimode interference
*n*_i_: Refractive index of the incident medium
*n*_t_: Refractive index of the transmitted medium
Si_3_N_4_: Silicon nitride
SiO_2_: Silica
TiO_2_: Titanium dioxide
*θ*_i_: Angle of incidence
*θ*_t_: Angle of anomalous refraction
*λ*_0_: Incident wavelength in vacuum

## References

[1] Yu N, Genevet P, Kats MA (2012). Light propagation with phase discontinuities: generalized laws of reflection and refraction. Science.

[2] Yu Y, Zhu AY, Paniagua-Dominguez R (2015). High-transmission dielectric metasurface with 2π phase control at visible wavelengths. Laser Photonics Rev.

[3] Zhou Z, Li J, Su R (2017). Efficient silicon metasurfaces for visible light. ACS Photonics.

[4] Shalaev MI, Sun J, Tsukernik A (2015). High-efficiency all-dielectric metasurfaces for ultracompact beam manipulation in transmission mode. Nano Lett.

[5] Li Z, Kim MH (2017). Controlling propagation and coupling of waveguide modes using gradient metasurfaces. Nat Nanotechnol.

[6] Staude I, Miroshnichenko AE, Decker M (2013). Tailoring directional scattering through magnetic and electric resonances in subwavelength silicon nanodisks. ACS Nano.

[7] Decker M, Staude I, Falkner M (2015). High-efficiency dielectric Huygens’ surfaces. Advanced Optical Materials.

[8] Yu P, Besteiro LV, Yong H et al (2018) Broadband metamaterial absorbers. Advanced Optical Materials 1800995 10.1002/adom.201800995, https://onlinelibrary.wiley.com/doi/10.1002/adom.201800995##

[9] Yu P, Besteiro LV, Wu J (2018). Metamaterial perfect absorber with unabated size-independent absorption. Opt Express.

[10] Ding F, Wang Z, He S (2015). Broadband high-efficiency half-wave plate: a supercell-based plasmonic metasurface approach. ACS Nano.

[11] Chen M, Cai J, Sun W (2016). High-efficiency all-dielectric metasurfaces for broadband polarization conversion. Plasmonics.

[12] Li T, Hu X, Chen H (2017). Metallic metasurfaces for high efficient polarization conversion control in transmission mode. Opt Express.

[13] Owiti EO, Yang H, Liu P (2018). Polarization converter with controllable birefringence based on hybrid all-dielectric-graphene metasurface. Nanoscale Res Lett.

[14] Sun S, Zhou Z, Zhang C (2017). All-dielectric full-color printing with TiO2 metasurfaces. ACS Nano.

[15] Zheng G, Mühlenbernd H, Kenney M (2015). Metasurface holograms reaching 80% efficiency. Nat Nanotechnol.

[16] Ee HS, Agarwal R (2016). Tunable metasurface and flat optical zoom lens on a stretchable substrate. Nano Lett.

[17] Chong KE, Staude I, James A (2015). Polarization-independent silicon metadevices for efficient optical wavefront control. Nano Lett.

[18] Gao S, Yue W, Park C (2017). Aluminum plasmonic metasurface enabling a wavelength insensitive phase gradient for linearly polarized visible light. ACS Photonics.

[19] Li Z, Palacios E, Butun S (2016). Ultrawide angle, directional spectrum splitting with visible-frequency versatile metasurfaces. Advanced Optical Materials.

[20] Li Z, Palacios E, Butun S (2015). Visible-frequency metasurfaces for broadband anomalous reflection and high-efficiency spectrum splitting. Nano Lett.

[21] Su Z, Chen X, Yin J (2016). Graphene-based terahertz metasurface with tunable spectrum splitting. Opt Lett.

[22] Aieta F, Genevet P, Kats MA (2012). Aberration-free ultrathin flat lenses and axicons at telecom wavelengths based on plasmonic metasurfaces. Nano Lett.

[23] Sun S, Yang K, Wang C (2012). High-efficiency broadband anomalous reflection by gradient metasurfaces. Nano Lett.

[24] Khorasaninejad M, Chen W, Devlin RC (2016). Metalenses at visible wavelengths: diffraction-limited focusing and subwavelength resolution imaging. Science.

[25] Zhan A, Colburn S, Trivedi R (2016). Low-contrast dielectric metasurface optics. ACS Photonics.

[26] Chen FT, Craighead HG (1995). Diffractive phase elements based on two-dimensional artificial dielectrics. Opt Lett.

[27] Hasman E, Kleiner V, Biener G (2003). Polarization dependent focusing lens by use of quantized Pancharatnam–Berry phase diffractive optics. Appl Phys Lett.

[28] Arbabi A, Horie Y, Bagheri M (2015). Dielectric metasurfaces for complete control of phase and polarization with subwavelength spatial resolution and high transmission. Nat Nanotechnol.

[29] Farmahini-Farahani M, Mosallaei H (2013). Birefringent reflectarray metasurface for beam engineering in infrared. Opt Lett.

[30] Guo Z, Zhu L, Shen F (2017). Dielectric metasurface based high-efficiency polarization splitters. RSC Adv.

[31] Guo Z, Zhu L, Guo K (2017). High-order dielectric metasurfaces for high-efficiency polarization beam splitters and optical vortex generators. Nanoscale Res Lett.

[32] Xu Y, Xiao J (2016). Compact and high extinction ratio polarization beam splitter using subwavelength grating couplers. Opt Lett.

[33] Zhang Y, He Y, Wu J (2016). High-extinction-ratio silicon polarization beam splitter with tolerance to waveguide width and coupling length variations. Opt Express.

[34] Guan X, Wu H, Shi Y (2014). Extremely small polarization beam splitter based on a multimode interference coupler with a silicon hybrid plasmonic waveguide. Opt Lett.

[35] Kim DW, Lee MH, Kim Y (2015). Planar-type polarization beam splitter based on a bridged silicon waveguide coupler. Opt Express.

[36] Lu Z, Wang Y, Zhang F (2015). Wideband silicon photonic polarization beam splitter based on point-symmetric cascaded broadband couplers. Opt Express.

[37] Palik ED (1985) Handbook of optical constants of solids. Academic Press: San Diego, pp 547–571

